# IS element IS*16 *as a molecular screening tool to identify hospital-associated strains of *Enterococcus faecium*

**DOI:** 10.1186/1471-2334-11-80

**Published:** 2011-03-31

**Authors:** Guido Werner, Carola Fleige, Uta Geringer, Willem van Schaik, Ingo Klare, Wolfgang Witte

**Affiliations:** 1 Robert Koch Institute, Wernigerode Branch, Wernigerode, Germany; 2 University Medical Centre, Utrecht, The Netherlands

## Abstract

**Background:**

Hospital strains of *Enterococcus faecium *could be characterized and typed by various molecular methods (MLST, AFLP, MLVA) and allocated to a distinct clonal complex known as MLST CC17. However, these techniques are laborious, time-consuming and cost-intensive. Our aim was to identify hospital *E. faecium *strains and differentiate them from colonizing and animal variants by a simple, inexpensive and reliable PCR-based screening assay. We describe here performance and predictive value of a single PCR detecting the insertion element, IS*16*, to identify hospital *E. faecium *isolates within a collection of 260 strains of hospital, animal and human commensal origins.

**Methods:**

Specific primers were selected amplifying a 547-bp fragment of IS*16*. Presence of IS*16 *was determined by PCR screenings among the 260 *E. faecium *isolates. Distribution of IS*16 *was compared with a prevalence of commonly used markers for hospital strains, *esp *and *hyl*_*Efm*_. All isolates were typed by MLST and partly by PFGE. Location of IS*16 *was analysed by Southern hybridization of plasmid and chromosomal DNA.

**Results:**

IS*16 *was exclusively distributed only among 155 invasive strains belonging to the clonal complex of hospital-associated strains ("CC17"; 28 MLST types) and various vancomycin resistance genotypes (*van*A/B/negative). The five invasive IS*16*-negative strains did not belong to the clonal complex of hospital-associated strains (CC17). IS*16 *was absent in all but three isolates from 100 livestock, food-associated and human commensal strains ("non-CC17"; 64 MLST types). The three IS*16*-positive human commensal isolates revealed MLST types belonging to the clonal complex of hospital-associated strains (CC17). The values predicting a hospital-associated strain ("CC17") deduced from presence and absence of IS*16 *was 100% and thus superior to screening for the presence of *esp *(66%) and/or *hyl*_*Efm *_(46%). Southern hybridizations revealed chromosomal as well as plasmid localization of IS*16*.

**Conclusions:**

This simple screening assay for insertion element IS*16 *is capable of differentiating hospital-associated from human commensal, livestock- and food-associated *E. faecium *strains and thus allows predicting the epidemic strengths or supposed pathogenic potential of a given *E. faecium *isolate identified within the nosocomial setting.

## Background

Vancomycin- and multi-resistant *Enterococcus *strains, especially strains of *E. faecium*, raise major concerns in intensive care medicine due to limited treatment options [1]. *E. faecium *is ecologically widely distributed and expected to play a central role as a reservoir and "turn-table" for antibiotic resistance determinants in the bacterial world, and especially among Gram-positive bacteria [2]. A number of molecular typing and characterization techniques such as AFLP, MLVA and MLST and comparative genomic hybridizations allowed differentiating strains of *E. faecium *and allocating them into various clonal complexes as based on core as well as accessory genome content [3,4]. Isolates belonging to hospital-associated clonal types (for instance, MLST CC17) could be identified and a supposed enhanced spreading potential among the nosocomial setting for isolates of this specific subgroup is predictable [4]. Reports from recent years described increasing annual rates of *E. faecium *bacteraemia in European countries [5,6]. When investigated in greater detail, the increase was due to increasing numbers of hospital-associated clonal types of *E. faecium *(MLST CC17) whereas numbers of other clonal types remained constant over time again emphasizing the aforementioned increased potential for nosocomial spread [5]. The molecular techniques to identify hospital-associated *E. faecium *strains are laborious, time-consuming and cost-intensive and are thus not applicable for routine diagnostics. Results of recent bacterial, epidemiological, microarray-based and genomic studies suggest that a number of markers are enriched among hospital strains of *E. faecium *including collagen adhesion factors *acm *and *scm *[7,8], other matrix-binding proteins and pili [9,10], a supposed hyaluronidase, *hyl*_*Efm *_[11,12], biofilm-associated markers including the enterococcal surface protein gene, *esp *[13,14], and various genomic islands encoding proteins of unknown functions [15,16]. Having acquired a number of these aforementioned determinants, these strains may have an increased pathogenic potential [4,10,17]. However, distribution of these markers among hospital strains is not exclusive and thus the predictive value is limited in terms of specificity and sensitivity. Certain markers possess pseudogenes and partly deleted variants complicating establishing a simple PCR based screening test [8,17]. Acquired ampicillin resistance appeared as a phenotypic trait of hospital strains, at least in Europe; however, this feature is also prevalent among non-hospital strains in some parts of the world, such as Asia and North America [18-20]. Microarray based genomic comparisons revealed a specific mobile element, insertion element IS*16*, exclusively prevalent among hospital *E. faecium *strains from an international collection of isolates [3]. In the present study, we established a PCR-based IS*16 *screening test and evaluated its robustness, sensitivity, specificity and predictive value for 260 pre-characterized isolates of *E. faecium *from different human and animal sources. IS*16 *was localized in a subset of *E. faecium *isolates (n = 45) representing 29 MLST types by Southern hybridization. Available *E. faecium *genome data were examined for the presence of IS*16 *homologous sequences and a genetic association of IS*16 *with genomic markers is discussed.

## Methods

### Strain collection

Our laboratory serves as a Focal Laboratory for Enterococci in Germany. In general, enterococcal isolates sent to us are pre-selected for being vancomycin- and multi-resistant or with the suspicion of clonal spread (local, regional, and country-wide outbreak analysis). Our collection of *E. faecium *test strains included 160 invasive isolates (blood cultures) from 60 different German diagnostic or hospital laboratories isolated between 1995 and 2007. Blood culture strains were pre-selected by geography and hospital origin, year of isolation, and different *van *genotypes (*van-/A/B*). The blood culture isolates were compared against 68 human commensal (outpatient) isolates from four different prevalence studies performed in different parts of Germany (1996, 1997, 1999, 2003) [24,25] and 32 isolates from animal (chicken, pigs) and meat samples (broiler, pork) (1994, 1995, 1999) [21-23]. In all cases duplicate isolates were excluded based on clinical/epidemiological data (same patient/hospital or animal sample/flock) and of molecular typing results (for instance, based on *Sma*I-macrorestriction using PFGE for human commensal isolates [24,25]; unpublished results). Antibiotic susceptibilities were determined by microbouillon dilution in MH bouillon. Ampicillin resistance is defined by an MIC of >8 mg/L and vancomycin resistance by >4 mg/L (http://www.eucast.org). Summarized data are presented in Table 1 and detailed information is given in Additional files 1, 2, 3. Ethical approval was not needed for this study; all strains were pre-selected and were received anonymously from German microbiological or hospital laboratories.

**Table 1:** Prevalence of epidemic markers among *E. faecium *isolates from different origins (see also Figure 1 and Additional Files 1, 2, 3).

Origin	No. of isolates	No. of MLST types	AmpR	PCR IS*16*+	PCR *esp*+	**PCR *hyl***_***Efm***_***+***	PCR *vanA+/B+*
Blood cultures	160	32	98.1% (n = 157)	96.9% (n = 155)	63.8% (n = 102)	45.6% (n = 73)	58.1% (n = 93)
*Hosp.-associated E. faecium (CC17)*^*1*^	*155*	*28*	*100% (n = 156)*	*100% (n = 156)*	*65.8% (n = 102)*	*46.5% (n = 72)*	*58.7% (n = 91)*
Human commensal	68	46	2.9% (n = 2)	4.4% (n = 3)^2^	0%	0%	14.7% (n = 10)
Animal commensal and meat sources	32	22	6.2% (n = 2)	0%	0%	0%	40.6% (n = 13)

BC MLST CC17 (second line) shown in italics (n = 155) is a subgroup of all blood culture *E. faecium *isolates presented in the first line representing all isolates allocated to the clonal complex of hospital-aascoiated strains (CC17).

AmpR, ampicillin resistance; ^1 ^including two isolates of ST65 representing a singleton but known to be found only among clinical strains; ^2 ^all three belonged to the clonal complex of hospital-associated strain types (MLST CC17; 2x ST18, 1x ST413; see also Additional file 4).

### DNA isolation

Genomic DNA was prepared using a DNA extraction kit (DNeasy Tissue Kit; Qiagen, Hilden, Germany) according to the manufacturer's instructions. An initial cell wall lysis step was added dissolving the cell pellet in TES buffer [10 mM Tris, 0.5 mM ethylene diamine tetra-acetic acid (EDTA), 10% sucrose (pH 8.0)] plus 10 mg/mL lysozyme (Roche Applied Science, Mannheim, Germany) followed by incubation at 37°C for 30 min. Plasmid DNA was isolated using a common phenol/chloroform-based alkaline lysis strategy [26].

### PCR

PCR was performed with illustra™ PuRe Taq Ready-To-Go™ PCR Beads (GE Healthcare, Freiburg, Germany) according to the manufacturer's instructions. Approximately 0.5 μL of isolated genomic DNA (ca. 10 ng) and primers (200 nM each) were added. Amplification of fragments representing the *esp*, *hyl*_*Efm *_and *vanA/B *genes was performed in a multiplex PCR as described elsewhere [27]. The following strains and plasmids were used as positive control samples: plasmid pRUM (IS*16*), plasmid pLG1 (*hyl*_*Efm*_); plasmid pIP816 (*vanA*; *E. faecium *BM4147), *E. faecium *U0317 (*esp*), and *E. faecalis *V583 (*vanB*). *E. faecalis *OG1RF served as a negative control sample for all PCR assays. PCR primers and conditions for screening IS*16 *elements were as follows: IS16-F: 5'-CATGTTCCACGAACCAGAG and IS16-R: 5'-TCAAAAAGTGGGCTTGGC, annealing temperature was 53°C, the predicted size of the product was 547 bp (reference *E. faecium *plasmid pRUM; GenBank acc. no. AF507977). Primers were established using software Acelrys DS Gene 1.5.

### PFGE typing

Genomic DNA for PFGE analysis was isolated and treated as described recently [27]. The agarose gel concentration was 1%, the CHEF-DR III apparatus (Bio-Rad Laboratories, Hercules, CA, USA) was used for PFGE. The ramped pulsed times were as follows: 1 - 11 sec for 15 h and 11 - 30 sec for 14 h at 14°C. *Sma*I-digested *Staphylococcus aureus *NCTC 8325 was used as a molecular mass standard on all PFGE gels. Chromosomal DNA of *E. faecium *strains was digested with I-*Ceu-I *for 16 h at 37°C. Plasmids were linearized by S1 nuclease (TaKaRa, Germany) treatment for 15 min (37°C) and subsequent resolution of the linearized plasmid DNA in PFGE. The ramped pulsed times for I-*Ceu-I *gels were 5 - 30 sec for 22 h and for S1 PFGE 5 - 35 sec for 22 h at 14°C [28].

### Southern hybridizations

Southern hybridization experiments were done as described elsewhere using a PCR-generated digoxigenin-labelled IS*16 *probe (DIG High Prime; Roche Applied Science, D), hybridization chemicals and equipment from commercial kits and according to recommendations of the manufacturer (Roche, Germany). Immunological detection was done as recommended using a chemiluminescent probe (CDP-Star™, Roche, D) and several readouts were taken at 10, 30, 60 and 120 min in a chemi-imager from Bio-Rad (Chemidoc XRS, Bio-Rad Labs., Hercules, US).

### MLST and DNA sequencing

PCRs amplifying the seven loci used for MLST were done according to the reference (http://efaecium.mlst.net/). Sequencing reactions were performed according to the manufacturer's recommendations for cycle sequencing of PCR products (Applied Biosystems, Germany). Sequence files were read, evaluated, aligned and compared to the reference set of alleles using sequencing software Lasergene 8.0 from DNA-STAR (SeqMan 8.0; EditSeq 8.0), TraceEditPro v. 1.1.1 from Ridom (http://www.ridom.de), and via the official MLST webpage (http://efaecium.mlst.net/). Phylogenetic trees were generated using software programmes eBURST [29] for the set of determined MLST types and global optimised eBURST [30] for the entire set of all available 524 MLST types (http://efaecium.mlst.net/).

### Statistics

Statistical analyses were performed with software package EpiCompare 1.0 (Ridom).

## Results

### Identification of IS*16 *primer sequences

Based on available sequences (pRUM [31] or composite (*vanB*) transposons [32]) we established PCR primers for an IS*16*-specific internal fragment (547 bp). Specificity and sensitivity was evaluated against available bacterial databases (BlastN, GenBank). The PCR was established using DNA from reference samples known to be positive for IS*16 *(plasmid pRUM) and from negative control samples known to lack IS*16 *(*E. faecalis *OG1RF). PCR results were as expected and amplified products from reference samples were sequenced for confirming specificity of the amplified PCR product (not shown).

### Screening for markers in the two sets of strains

Genomic DNA was screened by PCR using the established PCR primers for IS*16*, *esp *and *hyl*_*Efm*_. IS*16 *was highly prevalent among invasive *E. faecium *(n = 155/160; Table 1) and mainly absent (n = 3/100) from human commensal or animal and meat sources. The three IS*16-*positive commensal isolates were from humans. Other markers supposedly enriched among clinical or hospital-associated *E. faecium *isolates were also assessed: the *esp *gene was prevalent among 64% of all blood culture isolates and 46% of all invasive isolates were *hyl*_*Efm*_-positive (Table 1). None of the 100 non-clinical strains possessed *esp *or *hyl*_*Efm*_. Ampicillin resistance was prevalent among almost all clinical strains (98%). Two human commensal and two animal isolates were also ampicillin-resistant.

#### MLST typing

**Figure 1:**
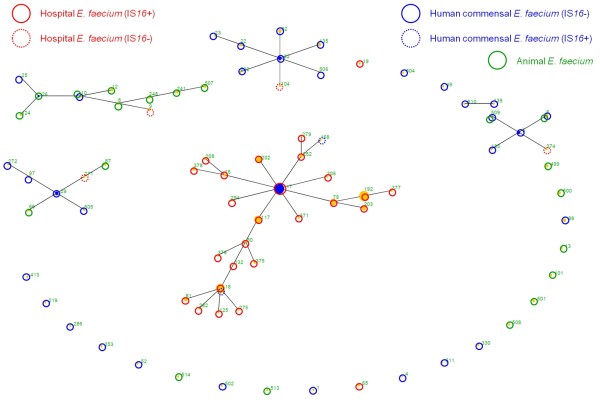
**eBURST cluster of MLST types generated from this set of 260 clinical, commensal and livestock-associated *E. faecium *strains**. (The newly identified MLST types are not presented as well as the following MLST types which appear as singletons in this set: ST39, ST74, ST94, ST289, ST345, ST503, ST413; ST512; compare also to Additional file 4). The origin of the isolates is given in colour codes. ST19 appears here as a singleton but belongs to CC17 when resolved among the entire set of MLST types (Additioinal file 4).

All 260 *E. faecium *isolates were MLST typed. Discriminatory power among clinical isolates (n = 160 isolates; 32 MLST types DI = 0.889 95% CI [0.864 - 0.913]) was much less than among human and animal commensal isolates (n = 100 isolates, 64 MLST types; DI = 0.988; 95% CI [0.983 - 0.994]). This suggests that prevalent hospital-associated clonal types differ less than their colonizing counterparts in animals and humans. In fact, 126 of the 160 invasive *E. faecium *isolates belonged to only eight MLST types known to represent major hospital-associated clonal types (ST16, ST17, ST18, ST78, ST117, ST192, ST202, ST203) whereas the other 24 MLST types occurred only occasionally. Based on their MLST types and subsequent eBURST and goeBURST analyses, isolates could be sorted into different clonal complexes which were in agreement with their corresponding ecological background (compare also to [4]). Figure 1 shows eBURST trees generated with all MLST types determined in this study. A phylogenetic tree of all currently detached MLST types (n = 524; 21.04.2010; http://efaecium.mlst.net/) using goeBURST is shown as Additional File 4. Based on these two calculations, almost all (n = 155/160) invasive isolates belonged to the clonal complex of hospital-associated strains (CC17 + ST65 [singleton]). The five IS*16*-negative clinical isolates represent colonizing strain types not associated with clonal types of hospital strains ("non-CC17"; ST9 [n = 2], ST104; ST271; ST374). The majority of commensal isolates represented many individual MLST types but belonging to the major clonal complexes of animal and human colonizing strain types (CC5, CC22/36, CC1). The three IS*16*-positive commensal *E. faecium *isolates revealed MLST types (ST18, ST413) belonging to the clonal complex of hospital-associated strains (CC17).

### Southern hybridizations of IS*16 *probes

The localization of IS*16 *among 45 representative *E. faecium *isolates from various origins and altogether 29 different MLST types was investigated. I-*Ceu-I *digested chromosomal DNA resolved in PFGE and plasmid DNA analysed inS1 PFGE and common agarose gels were transferred onto nylon membranes and hybridized against a labelled IS*16 *probe. Results are shown in Table 2. If available, several representatives of identical MLST types differing in their *van *gene content were considered for analysis (see for instance ST17 *vanA/B/*-negative isolates). In general, the IS*16 *probe hybridized to (up to three) chromosomal bands suggesting several copies of the same element present in the genome (a single hybridizing chromosomal band could still result from several IS*16 *copies on it). Four isolates (ST17, ST81, ST202, ST275) showed IS*16 *only hybridizing to plasmid bands. Twenty-two strains (n = 22/45) revealed a signal for both chromosomal as well as plasmid bands. Hybridization patterns were independent of the *van *gene content (Table 2).

**Table 2:** Localization of IS*16 *to I-*Ceu-I *digested chromosomal DNA resolved in PFGE and plasmid DNA as based on Southern hybridizations.

**Strain no**.	Origin	MLST type	*van *genotype	I-*Ceu*I PFGE	Plasmid
UW 6714	BC	**202**	-	-*	+
UW 1245	BC	**202**	*vanA*	-	+
UW 6293	BC	**202**	*vanB*	-*	-^#^
UW 6295	BC	**202**	*vanB*	-*	-^#^
UW 4962	BC	**203**	*vanA*	+	-^#^
UW 6989	BC	**203**	-	+	-^#^
UW 6502	BC	**208**	*vanB*	+	+
UW 5918	BC	**252**	*vanA*	+	+
UW 4675	BC	**252**	*vanB*	+	-
UW 6617	BC	**275**	*vanA*	-	+
UW 4960	BC	**279**	*vanB*	+	-
UW 6033	BC	**282**	*vanA*	+	+
UW 4512	BC	**294**	-	+	+
UW 6297	BC	**306**	-	+	+
UW 6935	BC	**371**	-	+	+
UW 3142	BC	**375**	*vanA*	+	+
UW 5275	BC	**376**	*vanB*	+	+
UW 6983	BC	**377**	-	+	+
UW 6112	BC	**378**	-	+	+
UW 4208	BC	**New**	-	+	+
UW 6642	BC	**New**	*vanA*	+	+
AK-EM 40	HC	**18**	-	+	+
AK-EM 53	HC	**413**	-	+	+
K226.7	HC	**18**	-	+	-
UW 2055	BC	**16**	*vanA*	-*	+
UW 3695	BC	**17**	-	+	+
UW 3544	BC	**17**	*vanA*	+	-^#^
UW 5352	BC	**17**	*vanB*	-	+
UW 6990	BC	**18**	-	+	+
UW 1952	BC	**18**	*vanA*	+	+
UW 2900	BC	**18**	*vanB*	+	-^#^
UW 1218	BC	**19**	*vanA*	+	-^#^
UW 3056	BC	**65**	-	+	+
UW 6880	BC	**78**	-	+	+
UW 4671	BC	**78**	*vanA*	+	-
UW 2457	BC	**80**	*vanA*	+	-
UW 6985	BC	**81**	-	-	+
UW 2771	BC	**81**	*vanA*	+	-
UW 6882	BC	**117**	-	+	-
UW 1983	BC	**117**	*vanA*	+	-
UW 6805	BC	**125**	*vanA*	+	+
UW 2824	BC	**132**	*vanA*	+	-^#^
UW 1716	BC	**192**	-	+	-^#^
UW 5852	BC	**192**	*vanA*	+	+
UW 5267	BC	**192**	*vanB*	+	-^#^

If available, different representatives of identical MLST types were considered (see for instance ST17 *vanA/B/-*negative isolates).

PFGE, pulsed-field gel electrophoresis; MLST, Multi-locus sequence typing; BC, blood culture; HC, human commensal; *, Hybridization signal with PFGE slot; ^#^, negative results in repeated experiments.

### Screening *E. faecium *genomes for IS*16*

Several *E. faecium *genomes are available as genomic fragments for preliminary analysis (collection I: seven *E. faecium *genomes; [33]; collection II: eleven *E. faecium *isolates from the Human Microbiome Project; (http://nihroadmap.nih.gov/hmp/; http://www.ncbi.nlm.nih.gov//genomes/geblast.cgi?gi=6511#SearchSet). A BLAST search for IS*16*-related sequences in both *E. faecium *collections revealed several hits. However, as far as some detailed information was available, the neighbouring DNA fragments all showed different genetic links. IS*16 *was prevalent among four out of seven genomes in collection I [33]. IS*16 *was linked to (a) several putative fragments of a conjugative transposon (strain E1162, ST17), (b) ORF involved in sugar metabolism (strain E1636; ST106), (c) a genomic fragment containing other transposases of the IS*256 *family (strain U0317, ST78) and (d) the supposed pathogenicity island containing the *esp *gene (strain E1679, ST104). None of the other two strains also containing the *esp *PAI possessed IS*16 *within its structure. In the deposited genomic fragments from the Human Microbiome Project, IS*16 *was infrequently identified, for instance, in *E. faecium *1,230,933 linked to a metal-dependent hydrolase or in *E. faecium *TC6 linked to an extracellular solute-binding protein, family 1. In *E. faecium *DO a 5' link to a C4-dicarboxylate anaerobic carrier gene and a 3' link to a chloramphenicol actetyltransferase resistance gene followed by a replicase and relaxase genes indicated a supposed plasmid origin of this contig (not shown in details). Available sequence data allow extracting the MLST type related allele information out of the sequenced genomes and a corresponding analysis was done on the seven (collection I) and eleven (collection II) accessible genomes. Of the 11 isolates positive for IS*16 *eight strains possessed hospital-associated MLST types (ST16, ST17, ST18, ST78, ST203, ST-NEW) and three did not belong to clonal complexes of hospital-associated types (ST25, ST104, ST114). Intriguingly, of the latter three, one was a clinical isolate also positive for *esp*, a marker commonly associated only with hospital-associated strains.

### Epidemiological analysis of clinical strains

We introduced PCR-based screening for IS*16 *for all *E. faecium *isolates sent to our Focal Laboratory for Enterocooci in Germany in late 2007. In 2008 we received 314 *E. faecium *isolates (from >57 hospitals located in 13 of the 16 German federal states; all VRE) and in 2009 334 *E. faecium *isolates (from >49 hospitals located in 11 German federal states, 90% VRE) from clinical sources for further analyses. Altogether 99.4% (n = 312/314) in 2008 and 97.3% (n = 325/334) in 2009 were IS*16*-positive demonstrating wide prevalence of multi-resistant (VRE), hospital-associated strain types in hospitals in Germany.

## Discussion

Early recognition of epidemic *E. faecium *strains is critical for a timely introduction of strategies to prevent and control their further spread. Hospital-associated clonal types of *E. faecium *often disseminate unrecognized among the nosocomial setting before and until first VRE cases suddenly appear [5,6]. But standardised methods for rapid diagnostics of these hospital-associated *E. faecium *strains are missing and modern, DNA sequence-based typing techniques are laborious, time-consuming and comparably expensive. Acquired ampicillin resistance [5,6,34] and high-level ciprofloxacin resistance [35,36] appear as phenotypic traits of hospital-associated strains. However, ampicillin resistance in animal or commensal enterococci is also prevalent to a certain extent [37], at least in many North American and Asian countries [38] and the predictive value of acquired ciprofloxacin resistance is limited since it accumulates over time and differently in various clonal types of hospital strains [36]. Several genetic markers, such as *esp*, *hyl*_*Efm *_or the *purK1 *allele (used as part of the MLST scheme) are not ubiquitous traits of all hospital-associated *E. faecium *strains and failure to detect them does not reliably indicate a strain with a limited spreading or pathogenic potential [27,34,36,39-41]. It was also shown recently that *hyl*_*Efm *_is plasmid-located [11,42], transferable in vitro into several non-hospital *E. faecium *strains and thus cannot be suggested to serve as a specific marker to predict a clonal background of a given strain [43]. A reliable and rapid molecular test to differentiate commensal from hospital-acquired strains is desirable; results from comparative genomic hybridizations and genome sequencing projects show promising candidate markers including the described insertion element IS*16 *[3].

According to our data, IS*16 *is highly specific for predicting hospital-associated strain types (Table 1; Additional files 1, 2, 3). Altogether 155 of the 160 blood culture isolates from 1995 to 2007 from >50 contributing laboratories or hospitals and representing 28 MLST types were all IS*16 *positive. The comparably high number of (a) participating hospitals/laboratories contributing strains, (b) blood culture strains representing many different MLST types and different *van *genotypes, and (c) isolated from over 13 years compensates for a geographical limitation of a strain collection that only included isolates from Germany. The presence of IS*16 *was independent of resistance gene clusters known to possess this element; only 19 isolates were *vanB*-positive (12%) and the remaining isolates were vancomycin susceptible (n = 67; 42%) or of the *vanA*-type (n = 74; 46%). Since it is known from previous plasmid analysis that IS*16 *could be associated with specific plasmid types (e.g., pRUM), we identified the location of IS*16 *in a representative set of *E. faecium *isolates (*vanA/B/*-negative; 29 MLST types; Table 2). Southern hybridizations revealed chromosomal, plasmid and both localizations for IS*16 *suggesting a somehow restricted mobile gene pool within hospital-associated clonal types. All but three investigated human commensal and all animal strains possessed MLST types different from hospital-associated strains; all but the three aforementioned strains were IS*16 *negative. The three human commensal isolates possessing IS*16 *revealed MLST types ST18 and ST413, allocating them to hospital-associated strains and again emphasising the excellent predictive value of this element for identifiying hospital-associated strain types. PCRs for *esp *and *hyl*_*Efm *_were negative for these three strains and one was ampicillin-resistant. Absence of *IS*16 among non-clinical *E. faecium *strains also suggests a very limited host range of IS*16*-bearing plasmids (e.g., pRUM-like) only among hospital-associated strains. Prevalence of hospital-associated *E. faecium *isolates among the general human population is low and more detailed information was only available in one of the three cases. This outpatient from a community surveillance study received beta-lactam treatment during the time of sampling which could be the reason for identifying an ampicillin- and multiresistant, epidemic strain here (association with a hospital stay was not documented).

An epidemiological analysis of 648 *E. faecium *clinical isolates sent to our Focal Laboratory for Enterococci in 2008/2009 from German hospital patients revealed that 98% (n = 637) were IS*16*-positive, demonstrating country-wide prevalence of hospital-associated strain types among German hospitals in recent years. This finding was supported by wide prevalence of acquired high-level ciprofloxacin resistance among the same strain collection serving as an additional marker for hospital-associated strains [36]. Admittedly, most of the strains from 2008/2009 were vancomycin-resistant showing in retrospect that hospital-associated strains of *E. faecium *and not commensal strain types accumulated in the nosocomial setting and acquired *vanA*- or *vanB*-type resistance determinants.

The observation that an IS element, IS*16*, is among the most specific markers to define hospital-associated strains [3] appears curious regarding the promiscuous nature of these elements to transpose between several genomic backgrounds within a cell and the possible horizontal mobilization in cis or trans by activation via conjugative elements and subsequent transfer into new hosts. It has been argued that acquisition of specific elements, especially IS*16*, has contributed to the ecological success of hospital-associated *E. faecium *strains allowing higher levels of genetic variability including easier acquisition of mobile genetic elements that may support lifestyle in new or changing environments or ecological niches like the hospital environment [3,44].

## Conclusions

Our data show that an IS*16*-based screening assay appears as the best molecular screening tool to differentiate colonizing *E. faecium *isolates from hospital-associated *E. faecium *strains that show an enhanced spreading potential in the nosocomial setting. The predictive value of an IS*16 *result is superior to the predictive strengths of established tests targeting molecular markers like *esp *or *hyl*_*Efm *_or phenotypic traits like acquired ampicillin or high-level ciprofloxacin resistance. Progress in comparative genome-based analysis will most probably reveal additional markers to identify hospital associated *E. faecium *isolates with an enhanced spreading potential in the nosocomial setting [15,33].

## Competing interests

The authors declare that they have no competing interests.

## Authors' contributions

IK manages and supervises the central enterococcal database and records all typing and characterization data from routine enterococcal diagnostics. UG performed standard PCRs, characterization and identification experiments. CF performed additional typing experiments such as PFGE, MLST and Southern hybridizations. WvS provided seven unpublished genome data and analyzed them regarding genetic linkages between IS*16 *and neighbouring regions. GW and WW supervised the study; GW selected the primer sequences, evaluated and calculated all the PCR, MLST, PFGE and hybridisation results. GW, IK and WW wrote the manuscript.

## Pre-publication history

The pre-publication history for this paper can be accessed here:

http://www.biomedcentral.com/1471-2334/11/80/prepub

## Supplementary Material

Additional file 1 **Detailed information on the 160 clinical *E. faecium *isolates**. The blood culture strains were part of this study and of Werner et al., [36]. "0" indicates absence, "1" indicates presence of the corresponding marker (*esp*, *hyl*_*Efm*_, IS*16*, *vanA/B/*0). Legend: AmpR, resistance to ampicillin; year, year of isolation. Click here for file

Additional file 2 **Detailed information on the 68 human commensal *E. faecium *isolates**. The human commensal strains were part of several community prevalence studies performed in different parts of Germany and at different time periods (see text for details). "0" indicates absence, "1" indicates presence of the corresponding marker (*esp*, *hyl*_*Efm*_, IS*16*, *vanA/B/*0). Legend: AmpR, resistance to ampicillin; year, year of isolation. Click here for file

Additional file 3 **Detailed information on the 32 animal *E. faecium *isolates**. The animal strains were part of several prevalence studies performed in different parts of Germany and at different time periods (see text for details). "0" indicates absence, "1" indicates presence of the corresponding marker (*esp*, *hyl*_*Efm*_, IS*16*, *vanA/B/*0). Legend: AmpR, resistance to ampicillin; year, year of isolation. Click here for file

Additional file 4 **goeBURST analysis of all MLST types of the *E. faecium *database (n = 524 STs; 21.4.2010**; http://efaecium.mlst.net/). Only the major, superior complex without singletons and smaller complexes is shown. A number of singletons from Figure 1 belong to clonal complexes such as ST19 which is part of the large cluster and related to CC17. Other complexes separated in Figure 1 (CC32, CC29) are linked here to form a main, superior cluster. ST65 remains a singleton and is the only IS*16*-positive ST not phylogenetically linked to the clonal complex of hospital-associated strains (CC17). Click here for file
