# Supplementary figures and images for: IS element IS16 as a molecular screening tool to identify hospital-associated strains of Enterococcus faecium
Source: BMC Infect Dis. 2011 Mar 31;11:80. doi: 10.1186/1471-2334-11-80 (PMC3076258; doi:10.1186/1471-2334-11-80)

## Slide 1
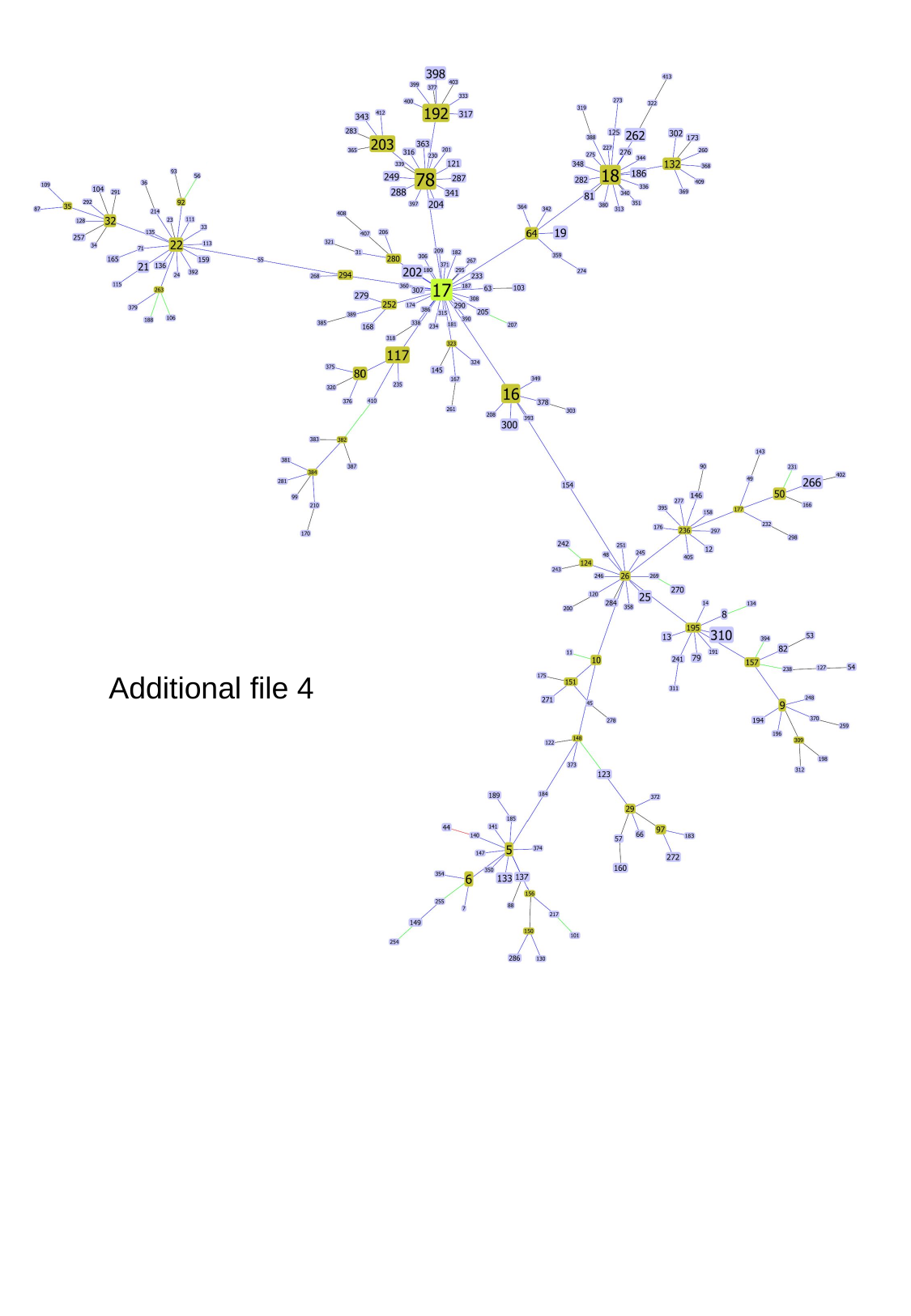

Additional file 4

Supplement: Additional file 4 — goeBURST analysis of all MLST types of the E. faecium database (n = 524 STs; 21.4.2010; http://efaecium.mlst.net/). Only the major, superior complex without singletons and smaller complexes is shown. A number of singletons from Figure 1 belong to clonal complexes such as ST19 which is part of the large cluster and related to CC17. Other complexes separated in Figure 1 (CC32, CC29) are linked here to form a main, superior cluster. ST65 remains a singleton and is the only IS16-positive ST not phylogenetically linked to the clonal complex of hospital-associated strains (CC17). [file 1471-2334-11-80-S4.PPT]
